# Public Health Implications of Latent Toxoplasmosis and Its Association With Type 2 Diabetes: A Case–Control Study in Qazvin, North‐Western Iran

**DOI:** 10.1002/hsr2.71782

**Published:** 2026-01-28

**Authors:** Leila Modarresnia, Meysam Olfatifar, Sayed Hussain Mosawi, Abouzar Babaei, Seyyed Hamidreza Ghafelehbashi, Ioannis Adamopoulos, Mohammad Ali Mohaghegh, Mehdi Bakht, Fariba Najar Hoseini, Ali Asghari, Aida Vafae Eslahi, Milad Badri

**Affiliations:** ^1^ Medical Microbiology Research Center Qazvin University of Medical Sciences Qazvin Iran; ^2^ Gastroenterology and Hepatology Diseases Research Center Qom University of Medical Sciences Qom Iran; ^3^ Medical Sciences Research Center Ghalib University Kabul Afghanistan; ^4^ Research, Institute for Prevention of Non‐Communicable Diseases Qazvin University of Medical Sciences Qazvin Iran; ^5^ Department of Public Health Policy, Sector of Occupational and Environmental Health, School of Public Health University of West Attica Athens Greece; ^6^ Department of Laboratory Sciences, School of Paramedical Sciences Torbat Heydariyeh University of Medical Sciences Torbat Heydariyeh Iran; ^7^ Health Sciences Research Center Torbat Heydariyeh University of Medical Sciences Torbat Heydariyeh Iran; ^8^ Department of Basic Medical Sciences Khoy University of Medical Sciences Khoy Iran

**Keywords:** case‐control study, diabetes mellitus, latent toxoplasmosis, seroprevalence, *Toxoplasma gondii*

## Abstract

**Introduction:**

*Toxoplasma gondii* is a worldwide‐distributed zoonotic parasite, causing latent infections in humans. Increasing evidence has suggested a possible link between toxoplasmosis and diabetes mellitus (DM), though data from Iran are limited. This study was conducted to determine the seroprevalence of latent *T. gondii* infection and its association with diabetes in Qazvin province, north‐western Iran.

**Materials and Methods:**

In this case‐control study, 350 patients with type 2 diabetes mellitus and 350 non‐diabetic controls were recruited from clinical laboratories in Qazvin province. Anti‐*T. gondii* IgG antibodies were detected by enzyme‐linked immunosorbent assay (ELISA). Data on sociodemographic variables and exposures were obtained by structured questionnaires. Statistical analyses, such as *χ* ^2^, *t*‐test, and logistic regression, were performed using the STATA software version 17.

**Results:**

Overall, 21.1% (148/700) of the participants were seropositive for *T. gondii* IgG antibodies. Compared with the controls, diabetic patients had a significantly higher seroprevalence of infection (27.7% vs. 14.6%). Seropositivity was positively associated with older age and rural residence, whereas no association was found with respect to sex, education, occupation, or dietary habits (consumption of raw or undercooked meat, raw eggs, and unwashed vegetables). Multivariate logistic regression, adjusted for age and residence, identified diabetes status as a significant factor associated with *T. gondii* seropositivity (adjusted OR = 2.62, 95% CI: 1.74–3.95, *p* < 0.001).

**Conclusions:**

The results indicated a higher seroprevalence of latent *T. gondii* infection in diabetic patients from Qazvin province, suggesting an epidemiological association between toxoplasmosis and diabetes. Significant correlates identified included age and living environment. These findings underscore a notable epidemiological link between latent toxoplasmosis and type 2 diabetes, highlighting the need for integrated public health attention to this association in endemic regions.

## 1 Introduction

*Toxoplasma gondii* is an obligate intracellular parasite of the phylum Apicomplexa, which infects humans and nearly all warm‐blooded animals. Toxoplasmosis affects an estimated one‐third of the world's population [1, 2]. Humans can acquire *T. gondii* through multiple pathways, such as ingesting oocyst‐contaminated food or water, consuming undercooked infected meat, vertical transmission, or via organ and blood transfusions [3, 4].

Acute *T. gondii* infection can be fatal in immunocompromised individuals, while chronic or latent infections may undergo reactivation in the context of immune system decline [5]. Most *T. gondii* infections are asymptomatic, but some individuals may develop clinical manifestations of toxoplasmosis, such as lymphadenopathy, chorioretinitis, or meningoencephalitis [6]. Although usually asymptomatic, primary infection can occasionally cause chorioretinitis or fetal injury when occurring during pregnancy [7].

In immunocompetent individuals, postnatal acquired *T. gondii* infection often goes unnoticed, but when symptoms do occur, they typically include cervical lymphadenopathy, fever, headache, joint pain, and fatigue. Following acute infection, *T. gondii* typically establishes latent infection, characterized by the formation of tissue cysts containing slow‐replicating bradyzoites in various organs [8, 9]. Latent toxoplasmosis affects 20%–80% of people, with prevalence shaped by factors such as the exposure to cats, geographic latitude, climate, sanitation, and eating behaviors [10].

The infection is mainly detected through serological testing, and in recent years, various laboratory methods have been introduced to distinguish between acute and latent infections. Numerous seroepidemiological studies have also been conducted [11]. During the latent phase of *T. gondii* infection, serum IgG levels peak within 2–3 months after initial infection and gradually decrease to a persistently elevated baseline [12]. Measurement of serum IgG is a standard approach for assessing population‐level seroprevalence of latent infection [13, 14].

Diabetes mellitus (DM) represents a significant global public health challenge in the 21st century, with projections estimating that 552 million people (7.7% of the population) will be affected by 2030 [15]. Diabetes, similar to conditions such as Human Immunodeficiency Virus (HIV) infection and other immunodeficiencies, can predispose individuals to opportunistic infections. Several studies have reported that people with diabetes are more susceptible to specific infections, including toxoplasmosis [16, 17, 18]. For instance, a study in Iran, the risk of toxoplasmosis infection among diabetic patients was approximately twofold higher than that observed in healthy controls (RR = 2.21; 95% CI: 1.6–3.7) [17]. Similarly, Modrek et al. reported positive IgG and IgM serological responses to toxoplasmosis were detected in 61% and 35.6% of diabetic patients, respectively [18].

Some literature highlights an ongoing debate about the relationship between toxoplasmosis and diabetes [19]. This debate has arisen because individuals with diabetes are more susceptible to opportunistic infections like toxoplasmosis [20]. Conversely, it has been proposed that toxoplasmosis may increase the risk of developing diabetes, given the ability of *T. gondii* to invade and multiply within pancreatic cells [21].

*Toxoplasma gondii* infection could increase susceptibility to type 2 diabetes (T2DM) diagnosis through reduction of β‐cells mass, pancreatic tissue necrosis, or pancreatitis. Insulin synthesis and release would be disrupted by the death or inflammation of beta cells in the pancreas, increasing the risk of T2DM [22, 23, 24]. Individuals with weakened immune systems often exhibit a reduced or delayed production of specific antibodies in response to an acute infection [25]. Patients with diabetes often exhibit compromised immune function, suggesting they may be more vulnerable to *T. gondii* infection [21, 24, 26].

Although several studies in Iran and other countries have reported an association between *T. gondii* infection and diabetes mellitus, most data originate from southern, central, and western regions of Iran, where toxoplasmosis seroprevalence ranges from 33% to 40% in the general population [27, 28], while diabetes prevalence is estimated at 11%–15% nationally and continues to rise [29]. Regional studies in provinces such as Tehran, Ilam, and Khuzestan have reported elevated *T. gondii* seropositivity among diabetic patients [17, 18, 30], but data from northwestern Iran, particularly Qazvin province, remain absent.

Qazvin province, located in the northwest of Iran, is a region with distinct climatic and socioeconomic characteristics that may influence both toxoplasmosis epidemiology and diabetes prevalence. Furthermore, while several studies in Iran have reported an association, many have not fully accounted for basic demographic confounders using multivariate analysis. Therefore, this case–control study was conducted to determine the seroprevalence of latent *T. gondii* infection and to assess its association with T2DM in Qazvin province, while adjusting for key available demographic and environmental factors such as age and residence. The study also aimed to evaluate other common risk factors for toxoplasmosis in this specific population.

## 2 Materials and Methods

### 2.1 Study Area, Participants, and Data Collection

A case‐control investigation was carried out between October 2024 and February 2025 among individuals referred to the clinical diagnostic laboratories in Qazvin province, Iran, for anti‐*T. gondii* IgG antibody test as requested by their physicians.

**Figure 1:**
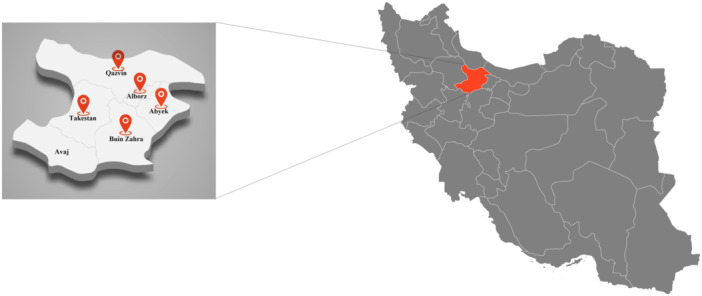
Georgraphic location of Qazvin Province, Iran (highlighted in red), showing its counties where diabetic cases and healthy controls were sampled for seroprevalence of latent *Toxoplasma gondii* infection.

Qazvin province in the northwestern part of Iran's central plateau is administratively divided into six counties: Abyek, Avaj, Alborz, Buinzahra, Takestan, and Qazvin [31, 32] (Figure 1).

The study population consisted of 350 individuals in the case group T2DM and 350 control individuals of various age groups. Cases and controls were frequency‐matched by age and sex to ensure comparable demographic distributions. Participants were considered healthy controls if they had no history of diabetes and their fasting and 2‐h glucose levels were within normal limits.

Each participant was asked to complete a structured questionnaire designed to collect the following information: age, sex, education level, profession, region, residential area, contact with animals, contact with soil, raw meat/raw eggs/vegetable consumption, source of drinking water, history of illness, and serological test results for anti‐*T. gondii* IgG.

After blood samples were collected from patients, they were informed about the research objectives and invited to participate in the study. All participants received complete information regarding the study purpose, procedures, and implementation methods before enrollment. The detection of anti*‐Toxoplasma* IgG in patient sera was performed using the enzyme‐linked immunosorbent assay (ELISA).

After obtaining informed consent, 5 mL of venous blood was collected from each participant under sterile conditions. The samples were centrifuged at 3000 rpm for 15 min to separate serum. The plasma samples were stored at −20°C, while the serum samples were kept at 2°C–8°C until testing. The ELISA test was performed according to the manufacturer's instructions provided with the commercial diagnostic kit (Pishtaz Teb Diagnostics, Iran).

The IgG titers were quantified based on optical density readings at 450 nm using an automated ELISA reader (Epoch, USA). Following the manufacturer's guidelines, 10 IU/mL served as the diagnostic cut‐off. Samples with antibody levels below this threshold were classified as seronegative, whereas those with titers equal to or above the cut‐off were regarded as seropositive.

According to the manufacturer's validation data (https://pishtazteb.com/en/products/elisa-kits/torch/toxoplasma-igg/), the Pishtaz Teb Anti‐*Toxoplasma* IgG ELISA kit demonstrates high diagnostic performance, with reported clinical sensitivity and specificity of approximately 100% when compared with reference serological methods. Assay precision was assessed using repeated measurements of positive and negative control sera, showing acceptable intra‐assay (within‐run) and inter‐assay (between‐run) coefficients of variation, generally below 10%–15%, indicating good repeatability and reproducibility. All assays were performed in accordance with standard laboratory quality control procedures, and kit controls were included in each run to ensure assay validity.

### 2.2 Statistical Analysis

In this study, STATA software, version 17 (StataCorp LLC, College Station, TX, USA), was employed for statistical data analysis. In this regard, alongside reporting the frequency and percentage of the studied variables based on the test results and in both case and control groups, analytical statistical methods were also applied.

Specifically, associations between qualitative variables were assessed using the *χ* ^2^ test, and differences in quantitative variables were analyzed using the *t*‐test. To further explore the data, logistic regression analysis was performed, including univariate analysis followed by a multivariate model to evaluate the relationships between variables.

Initially, univariate logistic regression analyses were conducted to estimate the crude odds ratios (ORs) for each independent variable, including group (case vs. control), age, sex, residential area, contact with animals, source of drinking water, contact with soil, and raw meat/raw eggs/vegetable consumption.

Variables with a *p* value less than 0.20 in the univariate analysis were entered into a multivariate logistic regression model to obtain adjusted ORs and 95% confidence intervals (CIs), controlling for potential confounding factors. A *p* value of < 0.05 was considered statistically significant.

## 3 Results

### 3.1 Demographic Characteristics of Study Participants

A total of 700 participants were included in this study, comprising 350 diabetic patients and 350 non‐diabetic controls. The mean age of participants in the case and control groups was 55.91 ± 12.64 and 55.60 ± 13.69 years, respectively, with no statistically significant difference between the two groups (*p* = 0.752). Age distribution showed that most participants in both groups were over 45 years old, with 42.9% of the diabetic and 37.4% of the control group aged above 60 years (*p* = 0.001).

The majority of participants in both groups (58.3% of diabetic patients and 64.9% of controls; *p* = 0.201) were females. Unemployment was the most prevalent occupational status in both groups, accounting for 64.0% of the diabetic group and 62.0% of the control group (*p* = 0.621). The most common level of educational attainment was primary education, reported by 50.6% of diabetic participants and 44.9% of controls (*p* = 0.237). The majority of participants were residents of Qazvin province (83.1% of diabetics and 80.6% of controls; *p* = 0.836). Urban residency was significantly more frequent among diabetic patients than among controls (94.0% vs. 84.3%, respectively; *p* < 0.001).

The detailed demographic characteristics of participants are presented in Table 1.

**Table 1:** Demographic characteristics of participants in the diabetic and control groups.

Variable		Case *n* (%)	Control *n* (%)	*p* value
Age range (years)	16–30	3 (0.9)	22 (6.3)	0.001
	31–45	83 (23.7)	85 (24.3)	
	46–60	114 (32.6)	112 (32.0)	
	> 60	150 (42.9)	131 (37.4)	
Sex	Female	204 (58.3)	227 (64.9)	0.201
	Male	145 (41.4)	122 (34.9)	
Occupation	Employee	47 (13.4)	44 (12.6)	0.621
	Housewife	75 (21.4)	81 (23.1)	
	Student	4 (1.1)	8 (2.3)	
	Unemployed	224 (64.0)	217 (62.0)	
Education	Illiterate	64 (18.3)	79 (22.6)	0.237
	Primary	177 (50.6)	157 (44.9)	
	University	109 (31.1)	114 (32.6)	
Province	Abyek	9 (2.6)	13 (3.7)	0.836
	Alborz	20 (5.7)	24 (6.9)	
	BoinZahra	4 (1.1)	3 (0.9)	
	Qazvin	291 (83.1)	282 (80.6)	
	Takestan	26 (7.4)	28 (8.0)	
Residential area	Urban	329 (94.0)	295 (84.3)	< 0.001
	Rural	21 (6.0)	55 (15.7)	

### 3.2 *Toxoplasma* IgG Seroprevalence and Related Risk Factors

Overall, 21.1% (148/700) of participants were seropositive for *T. gondii* IgG antibodies. The seroprevalence was significantly higher among diabetic patients compared with controls (27.7% vs. 14.6%, respectively; *χ*² = 18.13, *p* < 0.001).

Age was significantly associated with IgG seropositivity (*p* = 0.007), with the highest prevalence observed in participants older than 60 years (51.4%). In contrast, no significant associations were detected between IgG seropositivity and sex (*p* = 0.539), occupational status (*p* = 0.651), or level of education (*p* = 0.269).

The analysis revealed a significant association between residential area and *T. gondii* seropositivity (*p* = 0.003). Accordingly, among the 148 seropositive individuals, 26 (17.6%) resided in rural areas, while 122 (82.4%) were from urban areas.

Contact with animals, source of drinking water, soil contact, and consumption of raw meat, raw eggs, or unwashed fruits/vegetables were not significantly associated with *Toxoplasma* infection (*p* > 0.05).

A comprehensive summary of *Toxoplasma* IgG seroprevalence and its associated factors is provided in Table 2.

**Table 2:** *Toxoplasma* IgG seroprevalence and related risk factors among case and control groups.

Variable	Category	IgG positive *n* (%)	*p* value
Group	Case	97 (27.7)	< 0.001
	Control	51 (14.6)	
Age range	16–30	2 (1.4)	0.007
	31–45	26 (17.6)	
	46–60	44 (29.7)	
	> 60	76 (51.4)	
Sex	Female	87 (58.8)	0.539
	Male	61 (41.2)	
Education	Illiterate	33 (22.3)	0.269
	Primary	76 (51.4)	
	University	39 (26.4)	
Residential area	Rural	26 (17.6)	0.003
	Urban	122 (82.4)	
Contact with animals	Yes	29 (19.6)	0.179
	No	119 (80.4)	
Contact with soil	Yes	30 (20.3)	0.478
	No	118 (79.7)	
Source of drinking water	Unpurified	32 (21.6)	0.756
	Purified	116 (78.4)	
Raw meat/egg/vegetable consumption	Yes	74 (50.0)	0.638
	No	74 (50.0)	
History of illness	Present	71 (48.0)	0.805
	None	77 (52.0)	

The univariate logistic regression analysis identified significant associations between *T. gondii* IgG seropositivity and study group (case vs. control) (OR = 2.22, 95% CI: 1.52–3.24, *p* < 0.001), age (OR = 1.02, 95% CI: 1.01–1.04, *p* = 0.003), and residential area (OR = 2.02, 95% CI: 1.20–3.39, *p* = 0.008). In contrast, sex, contact with animals, contact with soil, source of drinking water, and consumption of raw meat, raw eggs, or vegetables were not significantly associated with seropositivity in the univariate model.

In the multivariate logistic regression analysis, after adjustment for potential confounders, study group (adjusted OR = 2.62, 95% CI: 1.74–3.95, *p* < 0.001), age (adjusted OR = 1.02, 95% CI: 1.01–1.04, *p* = 0.005), and residential area (adjusted OR = 2.41, 95% CI: 1.36–4.27, *p* = 0.002) remained independently associated with *T. gondii* IgG seropositivity. No statistically significant associations were observed for the remaining variables (Table 3).

**Table 3:** Logistic regression analysis of factors associated with *Toxoplasma gondii* IgG seropositivity.

Variable		Odds ratio (OR)	Std. error	*z*	*p* > |*z*|	95% CI
Univariate analysis	Group (case vs. control)	2.22	0.43	4.12	0.0	1.52–3.24
	Age (years)	1.02	0.01	2.99	0.003	1.01–1.04
	Sex (male vs. female)	1.18	0.22	0.86	0.39	0.81–1.70
	Residential area (rural vs. urban)	2.02	0.53	2.66	0.008	1.20–3.39
	Contact with animals	1.39	0.33	1.38	0.167	0.87–2.22
	Source of drinking water	0.94	0.21	−0.26	0.793	0.61–1.46
	Contact with soil	0.87	0.2	−0.62	0.536	0.56–1.35
	Raw meat/egg/vegetable consumption	1.1	0.2	0.52	0.601	0.77–1.59
Multivariate analysis	Group (case vs. control)*	2.62	0.55	4.61	0.0	1.74–3.95
	Age (years)*	1.02	0.01	2.83	0.005	1.01–1.04
	Sex (male vs. female)*	1.11	0.22	0.52	0.606	0.75–1.64
	Residential area (Rural vs. Urban)*	2.41	0.7	3.02	0.002	1.36–4.27
	Contact with animals*	1.45	0.39	1.41	0.159	0.86–2.45
	Source of drinking water*	0.86	0.2	−0.63	0.531	0.54–1.37
	Contact with soil*	1.03	0.26	0.12	0.901	0.63–1.69
	Raw meat/egg/vegetables consumption*	1.11	0.22	0.53	0.594	0.75–1.64

* Indicates variables that were statistically significant in the multivariable logistic regression model (*p* < 0.05).

**Figure 2:**
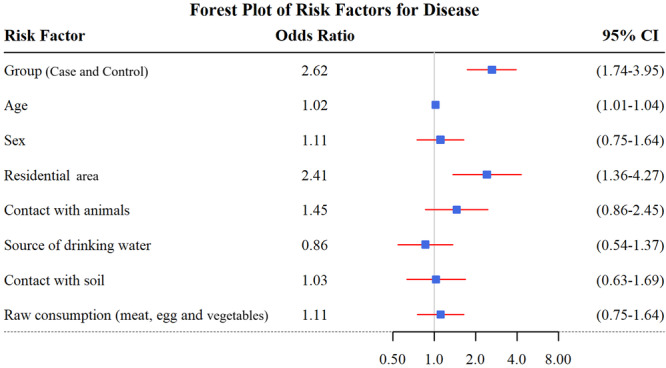
Forest plot of multivariate logistic regression showing odds ratios and 95% confidence intervals for variables associated with *Toxoplasma gondii* IgG seropositivity among diabetic and non‐diabetic participants.

Figure 2 shows the adjusted ORs and 95% confidence intervals for factors associated with *T. gondii* IgG seropositivity based on multivariate logistic regression analysis.

## 4 Discussion

This case–control study examined the association between latent *T. gondii* infection and T2DM in Qazvin province, north‐western Iran. Our findings indicate a higher seroprevalence of *T. gondii* IgG antibodies among diabetic patients compared to non‐diabetic controls, suggesting that latent toxoplasmosis may be more common in individuals with diabetes. Logistic regression analysis indicated that diabetes was significantly associated with *T. gondii* seropositivity even after adjusting for age and residential area, the key confounders available in our dataset.

Diabetic participants had more than twice the odds of *T. gondii* seropositivity compared to non‐diabetic controls. This association is consistent with previous studies in Iran and worldwide [17, 18, 30, 33, 34]. For example, Shirbazou et al. [17] found significantly elevated antibody levels among diabetic patients in Ilam province, and Soltani et al. [4] reported a similar trend in southwest Iran. Comparable findings have been documented in other regions of the world, including Egypt [34] and Iraq [19], further supporting the association between *T. gondii* infection and diabetes mellitus. A meta‐analysis by Majidiani et al. reported a pooled OR of 2.39 (95% CI: 1.20–4.75) for *T. gondii* infection in diabetic patients compared to controls [21]. Similarly, Catchpole et al. found an OR of 2.77 (95% CI: 2.03–3.76) indicates that individuals with T2DM had significantly higher odds of *T. gondii* seropositivity between 2.03 and 3.76 times those of the control group. [22].

However, when comparing our findings with previous studies, several factors must be considered. Studies from Iran and other countries have reported varying *T. gondii* IgG seroprevalence, which may reflect differences in sample size, population characteristics, study design, and the extent to which potential confounders were controlled. These methodological differences likely contribute to the heterogeneity in reported prevalence and underscore the importance of careful interpretation when comparing results across studies.

Several mechanisms can explain the observed association between *T. gondii* infection and diabetes: latent toxoplasmosis may result in inflammation and tissue destruction in the pancreas, leading to beta cell dysfunction and glucose intolerance through impaired insulin secretion [21, 24, 26]. On the other hand, DM may be an immunocompromised state predisposing patients to a higher risk of latent infections, including toxoplasmosis, due to impaired cell‐mediated immune responses [16]. This means that there may be a bi‐directional relationship, which indicates the importance of considering toxoplasmosis as a comorbidity in metabolic disorders.

Our results show that *T. gondii* seropositivity was significantly associated with older age, with the highest rates observed among individuals over 60 years. This pattern, commonly observed in serological studies [11, 13, 35]. These findings underscore the importance of targeted preventive measures for older adults, particularly those with diabetes.

The observed association between rural residence and higher odds of *T. gondii* seropositivity may reflect greater exposure to environmental sources of the parasite, such as soil or livestock, in rural areas. This finding aligns with previous studies reporting higher toxoplasmosis prevalence in rural populations [36, 37, 38].

In contrast to many earlier reports, our study did not find significant associations between *T. gondii* seropositivity and classical exposure routes such as direct animal contact, soil exposure, or consumption of raw meat, raw eggs, or unwashed fruits/vegetables. This discrepancy may be explained by several factors. First, improved food safety and hygiene practices in the region, particularly in urban and peri‐urban settings, may have reduced the risk associated with these behaviors. Second, our dichotomous (yes/no) assessment of exposures may have lacked the granularity needed to detect dose‐response relationships (e.g., frequency or intensity of exposure). Third, recall or social desirability bias in self‐reported behaviors could have attenuated true associations. Importantly, the persistence of a strong rural‐urban disparity despite the non‐significance of these specific factors suggests that other unmeasured environmental, infrastructural, or socio‐behavioral variables such as differences in soil contamination with oocysts, water sanitation systems, or food storage practices may be driving the higher rural seroprevalence in Qazvin province.

Sex, education level, and occupational status were not significantly associated with *T. gondii* seropositivity in our study. These variables have shown inconsistent associations in other studies, likely due to cultural and environmental differences affecting exposure risk [39, 40].

These data also contribute to the broader public health understanding of toxoplasmosis in Iran. Previous meta‐analyses estimated that latent *T. gondii* infection is carried by almost 33%–40% of the Iranian general population [27, 28], highlighting the need for integrated programs of infection control and education, with particular emphasis on the vulnerable groups such as patients with diabetes. The implementation of routine screening and preventive measures for ensuring food safety, improvement in water sanitation, and health literacy could reduce the disease burden. Future studies with multicenter participation, which incorporate molecular methods of diagnosis and longitudinal follow‐up, are required to help explain the temporal relationship and mechanisms between toxoplasmosis and diabetes.

The limitations of this study include the following: (1) the case–control design limits causal inference between *T. gondii* infection and diabetes; (2) due to the small number of type 1 diabetes samples (only two), this group had to be excluded from the analysis; (3) while we adjusted for key demographic factors, we did not collect data on other potential confounders such as body mass index (BMI), detailed socioeconomic indicators, specific dietary habits (e.g., frequency of raw meat consumption), direct exposure to cats, or the use of immunosuppressive medications. The absence of these variables means residual confounding cannot be ruled out, and our finding that diabetes is an independent predictor should be interpreted with this caution; and (4) information about behavioral and environmental risk factors was self‐reported and subject to recall bias.

## 5 Conclusions

This study found a significantly higher seroprevalence of latent *T. gondii* infection in diabetic patients compared to non‐diabetic controls in Qazvin province, Iran, with age and rural residence identified as key risk factors. These findings highlight latent toxoplasmosis as a public health concern for diabetic populations in the region. Therefore, it is recommended that healthcare providers consider integrating routine serological screening for *T. gondii* into the management plan for diabetic patients, particularly for older adults and those residing in rural areas. Coupling this with targeted patient education on preventive measures could help mitigate infection risk, prevent potential complications, and improve overall patient outcomes.

## Author Contributions

**Mehdi Bakht:** conceptualization, methodology, resources, data curation, writing – original draft, writing – review and editing, supervision, project administration, and funding. **Leila Modarresnia:** conceptualization, methodology, resources, and supervision. **Sayed Hussain Mosawi:** conceptualization, methodology, data curation, resources, and supervision. **Fariba Najar Hoseini:** resources and methodology. **Meysam Olfatifar:** formal analysis and data curation. **Seyyed Hamidreza Ghafelehbashi:** methodology and supervision. **Ioannis Adamopoulos:** methodology, data curation, writing – review and editing, and supervision. **Mohammad Ali Mohaghegh:** methodology, data curation. **Abouzar Babaei:** resources, methodology, and supervision. **Ali Asghari:** methodology and supervision. **Aida Vafae Eslahi:** methodology, writing – original draft, writing – review and editing, and supervision.

## Ethics Statement

This study received approval from the Institutional Review Board of Qazvin University of Medical Sciences, Qazvin, Iran, as recorded in the ethics documentation (no.IR.QUMS. REC.1403.401).

## Consent

All participants willingly agreed to participate in the testing process after receiving a full explanation of the study objectives and procedures. Written informed consent was obtained from every individual prior to sample collection and data inclusion.

## Conflicts of Interest

The authors declare no conflicts of interest.

## Transparency Statement

The corresponding authors, Meysam Olfatifar, Aida Vafae Eslahi, and Milad Badri, affirm that this manuscript is an honest, accurate, and transparent account of the study being reported; that no important aspects of the study have been omitted; and that any discrepancies from the study as planned (and, if relevant, registered) have been explained.

## Data Availability

The data that support the findings of this study are available from the corresponding author upon reasonable request.
